# Loss of Hierarchical Control by Occasion Setters Following Lesions of the Prelimbic and Infralimbic Medial Prefrontal Cortex in Rats

**DOI:** 10.3390/brainsci9030048

**Published:** 2019-02-26

**Authors:** Stephanie Roughley, Simon Killcross

**Affiliations:** School of Psychology, University of New South Wales, Sydney 2052, Australia; stephanie.kelly@unsw.edu.au

**Keywords:** prelimbic, infralimbic, medial prefrontal cortex, cognitive control, hierarchical control, occasion setting, extinction, Pavlovian

## Abstract

Recent work suggests complementary roles of the prelimbic and infralimbic regions of the rat medial prefrontal cortex in cognitive control processes, with the prelimbic cortex implicated in top-down modulation of associations and the infralimbic cortex playing a role in the inhibition of inappropriate responses. Following selective lesions made to prelimbic or infralimbic regions (or control sham-surgery) rats received simultaneous training on Pavlovian feature negative (A+, XA−) and feature positive (B−, YB+) discriminations designed to lead to hierarchical occasion-setting control by the features (X, Y) over their respective targets (A, B). Evidence for hierarchical control was assessed in a transfer test in which features and targets were swapped (YA, XB). All groups were able to learn the feature negative and feature positive discriminations. Whilst sham-lesioned animals showed no transfer of control by features to novel targets (a hallmark of hierarchical control), rats with lesions of prelimbic or infralimbic regions showed evidence of transfer from the positive feature (Y) to the negative target (A), and from the negative feature (X) to the positive target (B; although this only achieved significance in infralimbic-lesioned animals). These data indicate that damage to either of these regions disrupts hierarchical occasion-setting control, extending our knowledge of their role in cognitive control to encompass flexible behaviours dictated by discrete cues.

## 1. Introduction

In order to behave appropriately in environments that are complex and changing, operating via simple associative contingencies is often insufficient. Instead, an organism must be able to use task-relevant information to extract specific ‘rules’ for responding, as well as apply the top-down control necessary to implement and adapt these strategies as required, including both the capacity to promote relevant responses and the capacity to inhibit inappropriate responses. Previous research has implicated the medial prefrontal cortex (mPFC) in this sort of hierarchical control of behavioural responding, in recent years focusing specifically on prelimbic (PL) and infralimbic (IL) subregions. These regions appear to serve separable but complementary functions that together may offer an explanation as to how the mPFC might be organized to accommodate higher-order control processes. The research detailed below provides direct evidence of the involvement of PL and IL regions in the development of hierarchical control in the context of discrete cues controlling Pavlovian conditioning. This expands our understanding of the role of these areas in the flexible control of behaviour, complementing previous research examining contextual control of instrumental behaviours.

For example, in a rodent version of the Stroop task, work by Killcross, Haddon, and colleagues [1,2] provided explicit evidence for differential involvement of PL and IL cortices in hierarchical control of behaviour using contextual cues. Here, rats are trained on two biconditional discrimination tasks in separate contexts. These tasks involved presentations of two discriminable stimuli (auditory for one context: A1 and A2, Visual for the other: V1 and V2), each of which dictated that responding on a particular lever (L1 or L2) would be rewarded. At test, animals were presented with audio-visual compounds of these stimuli in each context, where either both stimuli had stipulated the same response in training (congruent) or had each stipulated an opposing response (incongruent). When presented with incongruent trials, animals are required to use the experimental context in order to disambiguate the conflicting response information and determine where to direct responding (i.e., the lever associated with the stimulus element that had been trained in that context). Results demonstrated that temporary inactivation of the PL cortex impaired the ability of these animals to perform context-appropriate responses on the incongruent trials, suggesting that the PL cortex, in particular, is necessary for using contextual information to guide appropriate responding in situations of cue and response conflict [2]. In contrast, using a version of the task in which the amount of training on the two discriminations was manipulated such that one discrimination received three times the training of the other [1], rats are normally unable to use the undertrained contextual cues to allow expression of the context-appropriate response when presented with an incongruent compound comprising both undertrained and overtrained cues. However, following inactivation of the IL cortex prior to the test, rats were able to overcome the impact of the overtrained discrimination in favour of the contextually appropriate undertrained cue. This suggests that the IL cortex usually functions to promote basic stimulus-response associations (which could be excitatory, inhibitory, or both), regardless of context, and oppose top-down modulation by the contextual cues.

The PL cortex has also been shown to be important for contextual control of responding in aversive Pavlovian conditioning [3]. In a contextual biconditional discrimination task, animals were trained with two cues in two contexts. Both cues were presented in both contexts, but in the first context, one cue was paired with a shock and the other with nothing, while in the second context this was reversed. Thus, the context dictated which cue to fear and which cue was safe. Inactivation of the PL cortex was found to interrupt both acquisition and performance of this discrimination, suggesting that the role of the PL cortex in hierarchical control processes extends to both appetitive and aversive domains. 

In both of the situations outlined above, each of the cues has a mixed associative history, sometimes preceding an appetitive or aversive outcome and sometimes not, and the context is required to determine which associative structure is operational at a given time. Another well-documented phenomenon that may involve the same sort of contextual modulation of simple associative structures is extinction. In extinction learning, an organism learns that a cue (CS) that previously predicted an outcome (US) no longer does, resulting in a decrease in conditioned responding to that stimulus. One prevalent model of extinction (Reference [4], but see also Reference [5]) stipulates that this learning can be thought of in terms of a new inhibitory association forming, which exists alongside the original excitatory one. This inhibitory association is context-dependent, such that it is only activated in preference to the original learning in contexts similar to that in which extinction learning occurred [6]. In this way, the context is said to gate the inhibitory association, modulating behavioural performance on the basis of which association is more likely to be valid in a particular environment.

There has been considerable research investigating the roles of PL and IL cortices in acquisition and expression of extinction learning. For example, the IL cortex has been shown to be important for the retrieval and expression of previously acquired extinction of fear learning [7], and lesions to the IL cortex have also been shown to increase recovery, reinstatement, and renewal of an extinguished Pavlovian response in an appetitive conditioning paradigm [8,9]. In contrast, lesions of the PL cortex have been shown to impair renewal of an extinguished fear response, with extinction learning appearing instead to generalize across contexts [10]. Taken together, these findings suggest that the IL cortex is important for facilitating the expression of extinction learning, and the generalization of inhibitory associations across contexts, while one possible function of the PL cortex is to gate the inhibitory extinction association on the basis of contextual features, modulating the expression of extinction based on contextual cues [11]. This is in line with evidence outlined above implicating the PL cortex in contextual modulation of behavior such that features of the organism’s current environment are better able to retrieve appropriate associations that come to be expressed in behaviour. Conversely, the IL cortex may be seen as again playing a complementary role by promoting the capacity of learning to be generalized across contexts, independently of higher-order cues or rules.

Many of these studies examining the role of PL and IL in the development of behavioural flexibility, or the implementation of rule-based strategies, have examined the ability of contextual cues to come to control performance. However, recent work has indicated that the PL and IL may also both play a role in the use of discrete cues to provide top-down control of response alternatives. On the one hand, Meyer and Bucci [12] have demonstrated that pre-training lesions of the PL region of the mPFC produce deficits in the acquisition of conditioned inhibition in a compound feature negative discrimination (of the form A+, AX−, where A+ is a 10-s tone followed by pellet reward, and AX- is presentation of a 10-s compound tone and light cue followed by no outcome). Rats with lesions of the IL region produced a level of discrimination that did not differ significantly from either PL-lesioned animals or sham-lesioned control animals. Prior to this, Rhodes and Killcross [13] examined the ability of rats with IL-lesions to acquire conditioned inhibition using a similar simultaneous feature negative design. As Meyer and Bucci also found, IL lesions were without effect in the acquisition of conditioned inhibition in this design, coming, like sham-operated control animals, to respond reliably more to A+ than AX− compounds. However, Rhodes and Killcross subsequently examined the status of the conditioned inhibitor X in both summation and retardation tests. In a summation test in which inhibitor X was paired with novel excitor B+, both lesioned and control animals showed an impact of the inhibitor X on responding, confirming that inhibition had accrued to X during training. In the retardation test, which examined the acquisition of conditioned responding to X (now X+) compared to a neutral cue (Y+) to which animals had had equivalent exposure, only sham-operated control animals showed the expected better acquisition to Y+ than to the inhibitory X+. For IL-lesioned animals, acquisition of conditioned responding was equal to both X+ and Y+. Rhodes and Killcross [13] concluded that the role of the IL cortex here was related specifically to the acquisition of both excitatory and inhibitory associations with the same cue, echoing its role in the inhibitory learning thought to occur in extinction, and more recent evidence suggesting a more general role in inhibitory associations [14]. 

However, these studies employing discrete-cue feature negative discriminations may not be directly comparable to those examining top-down modulation of behaviour by contextual cues, where the contextual cues are held to act as modulating factors. Whilst feature discriminations such as those described above are laid out as occasion-setting preparations, whereby the feature X can come to modulate the performance to the target A (much like the contextual cues may do in the studies outlined previously), evidence suggests that this is unlikely when simultaneous presentations of the cues in compound are used (as was the case in the above studies). Rather, simultaneous presentation of feature and target cues in compound (AX−) encourages the development of direct (inhibitory) associations between X and the target US or the behaviour engendered by presentation of A [4,15]. By contrast, however, modulatory relationships are favored when the feature and target compounds are presented sequentially or in series (that is, X followed by A). In these preparations, there is evidence that the feature X comes to modulate the relationship between A and the US (see, e.g., Reference [16]).

If sequentially-arranged feature discriminations are underpinned by occasion-setting relationships, the use of such procedures would seem a strong test-bed in which to further examine the role of PL and IL function. In particular, it would permit examination of both the development and execution of modulatory control over ambiguous relationships in discrete-cue Pavlovian conditioning in a manner that can be compared and contrasted with evidence derived from studies examining contextual modulation in both the Stroop analogue and extinction-based procedures. Such an addition would provide a broader base of evidence from which to derive conclusions about PL and IL function, specifically in the context of modulation of performance by top-down processes in which behavioural control must be exerted to regulate responding to cues with an ambiguous or mixed history of reinforcement.

Of course, as the argument above makes clear, simply observing the acquisition of feature discriminations does not, in itself, reveal the underlying associative structure; whilst we might arrange serial feature-target compounds to promote the development of hierarchical occasion setting control, this does not mean this is how the animals learn the tasks (they could, for example, nevertheless produce direct associations between features and the US). Whilst the precise associative structure underpinning occasion setting relationships are still somewhat in debate, in research that does evidence a hierarchical account over a configural one, the failure of complete transfer between occasion-setting cues plays a central role [4,16]. In general, features (X, Y) used in either sequential feature negative (A+, XA−) or feature positive (B−, YB+) discriminations do not show transfer of control over separately trained CSs (e.g., XC, or YD), and whilst there may be some (incomplete) transfer from one feature positive discrimination to another similarly trained feature positive discrimination, transfer does not occur between feature positive and feature negative discriminations under normal occasion setting circumstances [17]. The reason for this is that the modulation of the relationship between CS and US (whereby sometimes the CS predicts the occurrence of the US and sometimes it doesn’t) is found to be substantially CS and US specific, emphasizing that the feature modulates the relationship between CS and US, rather than the level of activation of associative representations of CS and/or US directly. Another factor made plain by this account is that the relationship between CS and US must be ambiguous (having a history of reinforcement and non-reinforcement) for occasion setting to play a strong role. Accordingly, a test of transfer provides a clear method by which to explicitly assess the associative structure underpinning feature discrimination.

The purpose of the current study is therefore two-fold. Firstly, we aimed to implement training of serial feature-positive and feature-negative discriminations and assess whether, under normal circumstances, animals acquire these discriminations via hierarchical occasion-setting mechanisms. Secondly, we aimed to examine the role of PL and IL cortices in this process—whether lesions of the PL or IL cortex impact acquisition of feature positive and feature negative discriminations, and/or the manner in which these are acquired. To achieve this, we trained rats (following excitotoxic lesions of PL, IL, or sham control surgery) simultaneously on both Pavlovian serial feature negative (A+ XA−) and Pavlovian serial feature positive (B+, YB−) discrete cue discriminations. Following acquisition of these discriminations, we examined the capacity of the features X and Y to transfer control to their oppositely trained targets (probe tests XB and YA in extinction). It is specifically by the use of the transfer task that one can reveal the specificity of the hierarchical control; if the training had brought the relationships between A and B and the US under hierarchical occasion setting control by X and Y respectively, then we would expect to see little or no transfer of control from X to B or Y to A. In contrast, if the normal processes of occasion setting were disrupted, we might expect this to be revealed in transfer of control from features X and Y to novel targets B and A.

If the PL cortex plays a general role in top-down control of both Pavlovian and instrumental tasks (as we have suggested, see Reference [11]), and the IL cortex plays a role in promoting and acquiring the basic associative relationships that are to be modulated, then the use of feature positive and feature negative occasion setting and transfer tasks should be revealing. It was expected that sham-operated control animals would demonstrate discriminative control of responding in both the feature positive and feature negative tasks, and furthermore would not show transfer of this control in the probe test. This would support the claim that discriminable responding to target cues in serially arranged occasion-setting preparations typically falls under hierarchical control via the feature cues. Given prior work indicating a role for the PL cortex in top-down modulation of performance towards ambiguous cues, one may expect PL-lesioned animals to show an impairment in learning the discriminations (for which there is some evidence in feature-negative preparations [18]). In addition, if they are able to learn the discrimination, it was expected that the transfer test would reveal this not to be a function of hierarchical control over responding (that is, these animals would show significant transfer). It is more difficult to come up with specific predictions as to whether and how lesions of the IL cortex may impact learning of the discriminations, since the main hypotheses regarding IL function are either firmly embedded in the context of extinction procedures [7] or relate to the development of stimulus-response associations [11] which do not have a clear translation in the context of Pavlovian conditioning. However, given the body of literature supporting dissociable functions of PL and IL regions of the cortex, it would be interesting to determine if a similar dissociation is observed in this paradigm. 

## 2. Materials and Methods

### 2.1. Subjects

Subjects for this experiment comprised twenty-four (*N* = 24) experimentally naive, adult male Long-Evans rats (Monash Animal Services, Gippsland, Victoria, Australia), weighing between 307–412 g at the start of experimentation. They were housed eight rats per cage, in a temperature- and humidity-controlled environment (22 °C) operating on a 12-h light-dark cycle (lights on at 7:00 a.m.). All experimental procedures took place during the light cycle. Following recovery from surgery, animals were placed on a food restriction schedule on which it was ensured they maintained at least 85% of free-feeding weight. Water was available ad libitum. All procedures were carried out in accordance with the National Institute of Health Guide for the Care and Use of Laboratory Animals (NIH publications No. 80-123, revised 1996) and were approved by the University of New South Wales Animal Care and Ethics Committee (ACE:09/39B).

### 2.2. Surgery

Prior to behavioral training, animals were randomly assigned to receive bilateral excitotoxic lesions of the infralimbic cortex, prelimbic cortex, or sham surgery (*n* = 8). Surgery was performed under isoflurane anesthesia in a standard stereotaxic frame (World Precision Instruments Inc., Sarasota, FL, USA), using a flat skull position. To produce lesions, 0.4 μL infusions of 10 μg/μL *N*-methyl-D-aspartic acid (NMDA; Sigma-Aldrich, Buchs, Switzerland) were administered to either the PL (coordinates from bregma; AP +3.2, L ± 0.7, and DV−4.0) or IL (coordinates from bregma; AP +3.0, L ± 0.7, and DV-5.4) cortex using a 1-μl syringe (Hamilton, NV, USA). Infusions proceeded at a rate of 0.1 μL/min, and once complete the syringe was left in place for a further four minutes to allow the solution to diffuse into the tissue. Animals in the sham group underwent an identical procedure, with the exception that no NMDA was administered. The syringe was entered at the PL site for four of the sham animals and at the IL site for the other four. Post-surgery all animals were allowed to recover over a minimum 10-day period, during which time they received daily post-operative observations, and had ad libitum access to both food and water. Animals were subsequently placed on the food restriction schedule, which was maintained for three days prior to the commencement of behavioural training and was continued for the duration of testing.

### 2.3. Apparatus

Training was carried out using eight standard operant chambers measuring 25 × 25 × 22 cm and housed in light- and sound-attenuating compartments (Paul Fray, Cambridge, UK). Each chamber was composed of three aluminum walls with a clear Perspex front wall and ceiling. The floors consisted of 18 stainless steel bars, 5 mm in diameter and spaced 1.5 cm apart. A magazine was located at the bottom center of the left-hand wall, where grain pellets (45 mg; Bio-Serv, Flemington, NJ, USA) could be delivered. Magazine entries were registered via the action of a Perspex flap that animals opened in order to access the magazine. Auditory stimuli were provided by a speaker fitted into the back center of the chamber’s ceiling, which was linked to an audio signal generator (Med Associates ANL-926, Fairfax, VT, USA). Auditory stimuli consisted of a 2.8 kHz tone and white noise. Visual stimuli consisted of a panel light located immediately above the magazine and an LED light located within the magazine itself. Experimental operations were controlled and recorded by a desktop computer equipped with MED-PC software (Med Associates Inc, Fairfax, VT, USA.).

### 2.4. Procedures

#### 2.4.1. Pretraining

Prior to discrimination training animals were given three 40-min sessions of magazine training, in which they learned to retrieve food pellets that were delivered to the magazine. Pellets were delivered approximately every 60 s according to a variable time schedule (VT60). For the first of these sessions only, the magazine flaps were fixed open to facilitate access to the rewards. 

#### 2.4.2. Discrimination Training

All groups received 32 sessions of discrimination training in which animals were exposed to concurrent serial feature positive and serial feature negative Pavlovian discrimination arrangements. In the feature positive arrangement, a visual ‘target’ stimulus (Magazine Light or Panel Light; designated cue B) was presented either alone with no reinforcement (B−) or was preceded by an auditory ‘feature’ stimulus (Noise or Tone; designated cue Y) and was accompanied by the delivery of a food reward (YB+). In the feature negative arrangement, the alternate target stimulus (Magazine Light or Panel light; designated cue A) was either presented alone, in which case the presentation was reinforced (A+), or was preceded by the alternate feature stimulus (noise or Tone; designated cue X) and was not reinforced (XA−). A summary of this experimental design is shown in Table 1. Combinations of auditory and visual stimuli were fully counterbalanced across animals. 

Each session ran for approximately 64 min and consisted of 10 stimulus presentations of each type (A+, XA−, B−, and YB+). In target-alone presentations, the target stimulus was presented for 10sec and was either followed by a reward or not, depending on trial type (A+ or B−). In feature-target presentations (XA− and YB+), the feature stimulus was presented for 10 s and was immediately followed by the 10 s target stimulus. For reinforced presentations, the termination of the target stimulus coincided with the delivery of a reward. These trials were presented in random order with the restriction of no more than two consecutive trials of the same type and were interspersed with variable inter-trial intervals (ITI; M = 60 s).

#### 2.4.3. Probe Test 

Following discrimination training, all groups were given a test session incorporating probe trials in which the original feature-target combinations were reversed. During this session groups received the discrimination procedure in its entirety, immediately followed by an additional 15 min in which animals were presented with six reverse feature-target presentations in the absence of reinforcement (three each of YA− and XB−). This was to assess whether the capacity of features X and Y to control responding would transfer to the opposing targets. These trials were interspersed with four more of the usual target-alone presentations (two each of A+ and B−). Trials were presented in random order and were separated by VT60 ITIs.

### 2.5. Histological Analysis

At the conclusion of behavioural testing, animals were given a lethal dose of sodium pentobarbitone and were transcardially perfused via the ascending aorta with saline-based pre-wash followed by 4% paraformaldehyde solution. Brains were removed and post-fixed in paraformaldehyde for a period of two days, before being transferred to sucrose solution (20% *w*/*v*) for a further 24 h. Forty micrometer coronal sections of the brain were taken using a cryostat and mounted onto gelatine-coated slides. Slides were air-dried overnight under a fume-hood and subsequently stained using cresyl violet. Lesion placement was verified under a light microscope, with the extent and location of neuronal damage for each animal recorded with reference to Paxinos and Watson’s atlas [19].

### 2.6. Statistics Analysis

Entry to the food magazine was the conditioned response measure of interest. Relative rates (per 10 s) of conditioned responding were calculated by subtracting average baseline levels of magazine entry from magazine entries performed during stimulus presentations. Baseline responding was defined as the rate of magazine entry during the ITI period, averaged across all trials of the session. Responding was measured over the full 10 s of feature presentations, and the final 5 s of target presentations (to minimize interference from behavioral competition on trials on which the feature preceded the target). Of primary interest were the rates of conditioned responding to target stimuli (A and B) during acquisition and the probe test. Data were analyzed using mixed analysis of variance (ANOVA) and where relevant, significant interactions were followed up with simple effects analysis and/or pairwise comparisons.

## 3. Results

### 3.1. Histology

**Figure 1:**
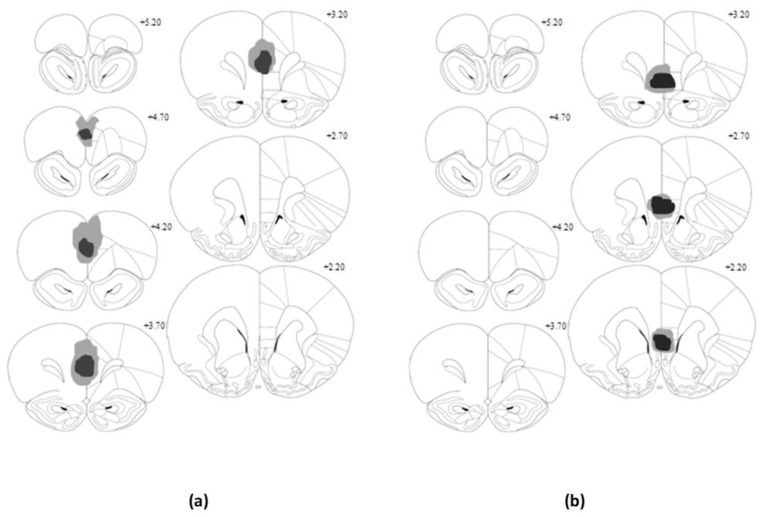
Schematic representation of location and extent of excitotoxic lesion damage to the prelimbic (PL; **a**) and infralimbic (IL; **b**) cortices. Shaded regions indicate the minimum (black) and maximum (grey) area covered by the lesions. Coronal sections displayed here are +2.20 to +5.20 (in mm from bregma).

All animals in the IL group showed substantial bilateral damage to the IL region, extending throughout anterior and posterior regions, while neighboring cortical regions were left largely intact (*n* = 8). Similarly, animals in the PL group showed acceptable levels of neuronal loss to the PL region, which extended fully in the anterior direction but showed some sparing of the posterior region (*n* = 8). Figure 1 illustrates the maximum (grey) and minimum (black) extent of lesion damage for both IL and PL groups. Location and extent of lesions in this study are similar to those in previous work, in which dissociable behavioural effects have been demonstrated [8,9,10,12,18,20]. 

### 3.2. Behaviour

#### 3.2.1. Acquisition

**Figure 2:**
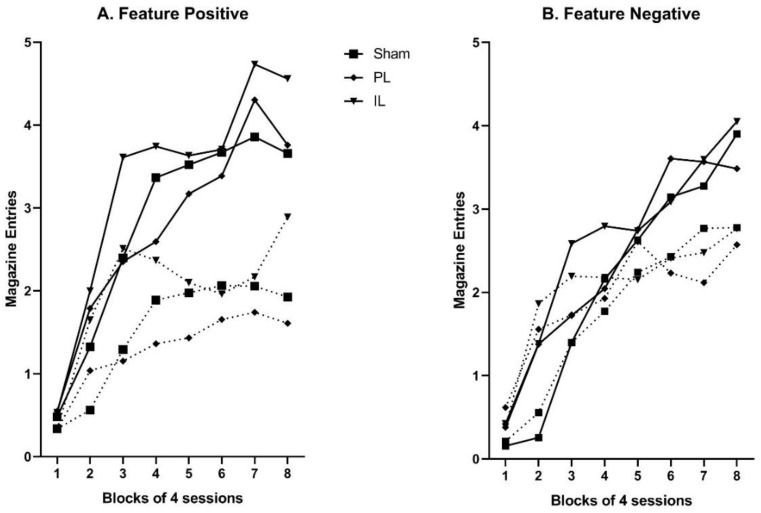
Acquisition of magazine entry responding (rate per 10 s) during target cue presentation in the feature positive (panel **A**) and feature negative (panel **B**) discriminations. Solid line displays responding to the reinforced target (i.e., on (Y)**B**+ trials for feature positive and **A**+ trials for feature negative), while dotted line displays responding to the non-reinforced target (i.e., **B**− trials for the feature positive and (X)**A**− trials for feature negative).

A preliminary analysis of baseline rates magazine entry was performed using a two-way mixed ANOVA in which the between subjects factor was Group (Sham, PL, and IL) and the within subjects factor was Session Block (1–8; blocks of four sessions). Results revealed no significant between-group differences in rates of baseline responding (*F* < 1). Similar ANOVAs (with additional within-subjects factor of Feature; present or absent) were also performed to analyze rates of acquisition of conditioned magazine entry responding to target stimuli in the feature positive (B alone vs. B when preceded by Y) and feature negative (A alone vs. A when preceded by X) discriminations. The data for these analyses are displayed in Figure 2. As illustrated, acquisition of the feature positive association is evidenced by increasing responding on reinforced YB+ trials compared to non-reinforced B- trials, and rate of acquisition did not differ by group. There were significant main effects of Session Block (*F*_7,147_ = 21.42; *p* < 0.001) and Feature (*F*_1,21_ = 27.74; *p* < 0.001) plus a significant Session Block by Feature interaction (*F*_7,147_ = 12.19; *p* < 0.001), but no other effects or interactions were significant (all *F* < 1). Acquisition of the feature negative association is evidenced by increasing responding on reinforced A+ trials, compared to non-reinforced XA− trials. Although it appears that animals in the PL lesion group may have acquired this discrimination more slowly than either the IL lesion or Sham control group (discriminated responding first appears in Block 3 or 4 for IL and Sham groups, but not until Block 6 for the PL lesion group), this was not statistically supported. As for the feature positive discrimination, there were significant main effects of Session Block (*F*_7,147_ = 15.13; *p* < 0.001) and Feature (*F*_1,21_ = 9.68; *p* = 0.005), as well as a Session Block by Feature interaction (*F*_7,147_ = 9.35; *p* < 0.001), but no other effects or interactions were significant (notably no main effect of Group or interaction with Group; all *F* < 1).

**Figure 3:**
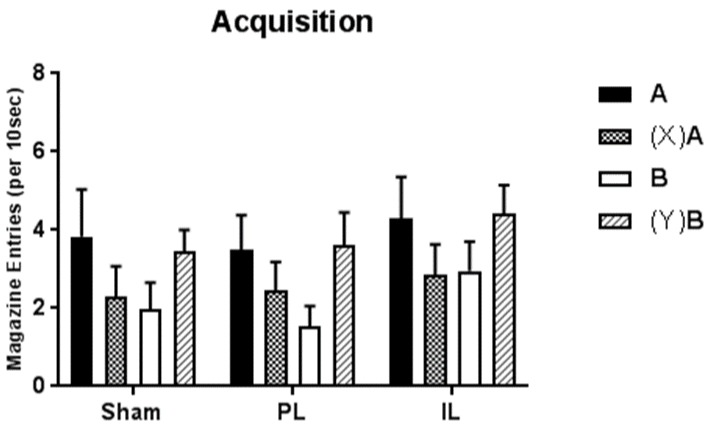
Rate (per 10 s; relative to baseline) of magazine entry during presentation of the target cue on feature-present and feature-absent trials of the feature negative ((X)A and A, respectively) and feature positive ((Y)B and B, respectively) discriminations. Error bars represent +SEM.

Responding to target stimuli during the final session of discrimination training is displayed in Figure 3. Rats in all groups showed evidence of having acquired both the feature positive and feature negative discriminations by the end of training, in that conditioned responding was greater on reinforced trials (A+ and (Y)B+) than non-reinforced trials ((X)A− and B−).

Acquisition of conditioned responding to target stimuli in the feature positive (B− and (Y)B+) and feature negative (A+ and (X)A−) discriminations was assessed via a three-way mixed ANOVA. In this analysis, the between subjects factor was Group (Sham, PL, and IL), and within subjects factors were Discrimination (feature negative or feature positive) and Feature (present or absent). Results revealed a significant Discrimination by Feature interaction (*F*_1,21_ = 29.88; *p* < 0.001). No other main effects or interactions were significant (all *F* < 1). 

Follow-up simple effects analysis of the interaction term indicate that averaging across groups, conditioned responding during target presentation on the feature negative discrimination was greater on the reinforced feature-absent trials (A+) than the non-reinforced feature-present trials ((X)A−; *F*_1,21_ = 12.57; *p* = 0.002). For the feature positive discrimination, conditioned responding during target presentation was greater on the reinforced feature-present trials ((Y)B+) than the non-reinforced feature-absent trials (B−; *F*_1,21_ = 18.55; *p* < 0.001). In other words, animals were able to use the feature cue to either inhibit (in the case of the feature negative discrimination) or elevate (in the case of the feature positive discrimination) responding to the target cue, and this did not differ as a function of lesion group. 

Analysis of responding during feature presentation was also assessed, using a two-way mixed ANOVA in which the between subjects factor was Group (Sham, PL, and IL) and the within subjects factor was Feature (X or Y; negative and positive discriminations, respectively). Results revealed a significant main effect of Feature (*F*_1,21_ = 24.86; *p* < 0.001), whereby conditioned responding during the positive feature Y (which is followed by reinforced target B; M (±SD) for Sham = 3.58 (±2.03), PL = 3.01 (±3.15), and IL = 2.86 (±2.64)) was greater than responding during the negative feature X (which is followed by non-reinforced target A; M (±SD) for Sham = 0.51 (±0.62), PL = 0.64 (±0.26), and IL = 0.54 (±0.49)). No other effect or interaction was significant (both *F* < 1), indicating that the ability to discriminate between excitatory (Y) and inhibitory (X) features did not differ as a function of lesion group. 

#### 3.2.2. Probe Test

**Figure 4:**
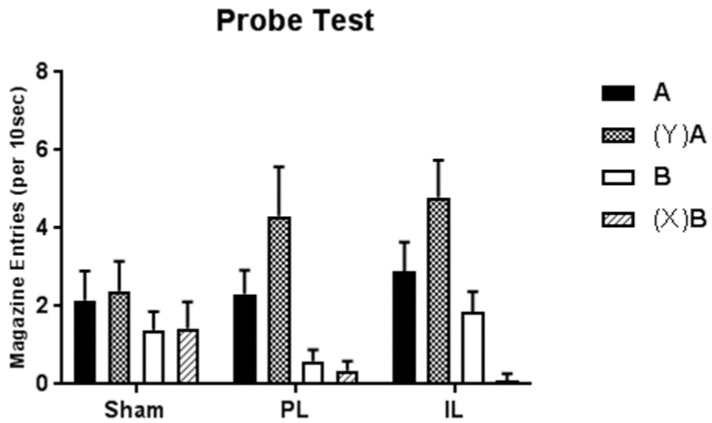
Rate (per 10 s; relative to baseline) of magazine entry during presentation of the target cue on feature-present ((Y)A and (X)B) and feature-absent (A+ and B−) trials of the probe test. Note paired features are the reverse of those in training. Error bars represent +SEM.

Rates of responding to target stimuli during probe trials are displayed in Figure 4. As illustrated, animals in both the PL and IL groups demonstrated differential responding to the target cues according to whether the cues were presented alone (A+ or B−) vs. when they were preceded by the feature opposite to that which they had been paired with in training ((Y)A or (X)B). Specifically, responding to A was increased when preceded by Y, and responding to B was decreased when preceded by X. In contrast, the presence of the reverse features did not appear to have a substantial effect on responding to target stimuli for animals in the Sham group. 

These findings are supported by a three-way mixed ANOVA in which the between subjects factor was Group (Sham, PL, and IL) and within subjects factors were Target (responding to A or B) and Feature (present or absent; Y and X, respectively). Results revealed a main effect of Target (*F*_1,21_ = 21.35; *p* < 0.001), whereby responding was greater during presentation of target cue A than target cue B, irrespective of feature presence or absence, and averaged across lesion group. This reflects the history of reinforcement for A and B alone (A+ and B-), and of X and Y as inhibitory and excitatory modulators respectively. Main effects of Feature and Group were not significant (*F*_1,21_ = 2.61; *p* = 0.12 and *F* < 1, respectively). 

More informative, however, is a significant three-way interaction between Group, Feature and Target (*F*_2,21_ = 4.09; *p* = 0.03) and two-way interaction between Feature and Target (*F*_1,21_ = 16.75; *p* = 0.001). Neither of the other interactions was significant (Group x Feature, *F*_2,21_ = 1.31; *p* = 0.29; Group × Target, *F*_2,21_ = 1.97; *p* = 0.16). Simple effects analysis clarifies the interaction terms, revealing that responding to target cue A was significantly greater when preceded by feature cue Y than when presented alone for both the PL and IL groups (*F*_1,21_ = 9.60; *p* = 0.005 and *F*_1,21_ = 8.59; *p* = 0.008, respectively), but not for the Sham group (*F* < 1). In addition, responding to target cue B was significantly lower when preceded by cue X than when presented alone for the IL group (*F*_1,21_ = 12.05; *p* = 0.002), though not for the PL or Sham groups (both *F* < 1). In the case of the PL group, however, this is likely to be a floor effect given responding was already minimal to B when presented alone. 

Together, these results indicate that both PL- and IL-lesioned animals demonstrate transfer of feature properties to different targets to a greater extent than sham-operated control animals. Specifically, in both these groups there is evidence of additive excitatory stimulus components to both A and Y, and in the IL group there is evidence of additive inhibitory stimulus components to both B and X. While this latter effect is not statistically supported in the PL group, it appears from that this may be a floor effect, in that responding was already minimal to B when presented alone.

## 4. Discussion

Here we have described a single experiment designed to examine the role of PL and IL regions of the mPFC in the development of hierarchical control in discrete cue Pavlovian feature negative and feature positive discriminations. In sham-operated control animals, simultaneous acquisition of the two discriminations proceeded uneventfully; although training was protracted, performance at the end of training indicated good control of performance in both discriminations, with significantly more responding to the rewarded cues (A+, and B+ following Y) than to non-rewarded cues (A− following X, and B−). A very similar pattern was observed in animals that had received lesions of the PL and IL prior to training, and no significant differences in acquisition were observed.

However, as indicated in the Introduction, performance during acquisition of these discriminations does not necessarily reveal the underpinning associative structure. As such we examined transfer of feature control over targets in separate probe tests. As expected (given the parameters used in the study such as the serial presentations of features and targets [17]), sham-lesioned control animals showed no transfer whatsoever across feature negative and feature positive discriminations. That is, probe presentations of positive feature Y prior to previously rewarded A failed to produce any significant enhancement of performance; similarly probe presentations of negative feature X prior to previously non-rewarded B failed to produce any significant decrement in performance. As such, we would conclude that in these sham-lesioned animals, the performance of the feature positive and feature negative discriminations was very likely to be the product of an underpinning hierarchical relationship in which the features came to modulate conditioned responding to targets with a mixed history of reinforcement. 

In contrast, discrete lesions of either the PL or IL resulted in probe test performance that was markedly different. In the case of IL lesions, presentations of the positive feature Y enhanced performance to novel target A, and presentations of the negative feature X attenuated conditioned responding to novel target B. A very similar pattern of responding was observed following PL lesions, although the decrement in conditioned responding to cue B following presentations of negative feature X failed to reach significance in post-hoc analyses. It is possible that this could simply reflect a floor effect as baseline responding to target B (in the absence of X) was especially low in this group of animals.

As such, one would have to argue that the control of performance in the feature positive and feature negative discriminations in groups with lesions of the PL or IL regions, whilst superficially similar to that of the sham-operated control group, was not controlled by the same underpinning occasion setting structure. Rather, the evidence of both positive and negative transfer from features to targets suggests that instead, the features have come to control performance by something other than a hierarchical modulation of the association between targets and rewards. Based on alternative findings from the occasion setting literature investigating the potential associative structures that may control feature positive and feature negative discriminations (see, e.g., References [4,16]), it seems likely that the features X and Y had developed their own associations with the US (inhibitory and excitatory, respectively), and so came to control performance by a process of summation with separate associations formed between targets A and B with the US (excitatory and inhibitory, respectively). Accordingly, acquisition of the discriminations in lesioned groups may be understood in terms of straight-forward error-correction models of associative learning (in particular the Rescorla-Wagner model [21]), whereby excitatory and inhibitory associations of features and targets with the US sum to control performance. Briefly the Rescorla-Wagner model posits that associative learning occurs by a process of error correction whereby the outcome expected to occur following presentation of a stimulus is compared to that which actually occurs and the difference—the prediction error—is then used to adjust the expectancy when the stimulus is next encountered. A key tenet of this model is that associative strength (or in other words, outcome expectancy) accrues separately to all stimuli present on a trial, and is additive; hence the expectancy that is generated when a number of stimuli are presented is equal to the sum of the expectancy for each stimulus (for example inhibition accruing to X can offset excitation accruing to A, leading to lower expectancy on XA trials and hence to lower CRs). 

Much previous work has implicated a role for the PL region of the mPFC in the top-down control of behavior. Inactivation of the PL cortex impairs performance in a rodent analogue of the Stroop task [2] and a contextual biconditional discrimination task [3], and PL lesions impair renewal of an extinguished fear response [10] and acquisition of a compound feature negative discrimination [12]. In addition, of particular relevance to this study MacLeod and Bucci [18] found evidence for a modest impairment in the rate of acquisition of a serial feature negative discrimination following lesions targeting the PL region (but not following lesions of the IL region). Whilst there were no statistically significant differences in acquisition between the three groups in the present study, there was some indication that learning of the feature negative discrimination may have been slower in the PL-lesioned group; rats with PL lesions failed to respond differentially to A+ and AX− until about the 6th block of training, compared to about the 3rd or 4th in Sham and IL-lesioned rats. Combined with the data showing transfer of occasion setting control, we would suggest that—as expected—lesions of the PL mPFC lead to a disruption of the ability of feature cues within occasion setting procedures to act as top-down modulators of associations formed between the target and US. Whilst rats with PL lesions (as we have observed in other Pavlovian procedures [3]) appear to remain able to call upon learning processes that conform to a Rescorla-Wagner model of associative learning [21], they cannot use cues or contexts in a hierarchical manner to modulate performance towards ambiguous stimuli with a mixed history of reinforcement. In this way, the data presented here confirm conclusions from previous work, as well as extending those findings to encompass the role of the PL region in the use of discrete stimuli as hierarchical modulators.

By contrast, the impact of lesions on the IL region on the acquisition and transfer tests are somewhat more surprising. Whilst we found no evidence at all for any deficits in acquisition of the two discriminations, both the positive and negative features were shown to be capable of transfer of control to novel targets, closely aligning with the observations following lesions of the PL region. In previous work, the impact of manipulation of PL and IL regions has tended to produce clearly dissociable results, often described as providing complementary functionality that collectively supports the appropriate implementation of flexible, task-appropriate behaviour. As discussed previously, while the PL cortex has been implicated in expression of Pavlovian conditioned associations, the IL cortex is implicated in the expression of extinction learning [7,22]. Furthermore, while substantial evidence exists to implicate the IL region in inhibitory control [23,24], the PL cortex does not appear to be involved in this operation, being implicated in perseverative rather than premature responding [25]. Marquis et al. [2], Haddon and Killcross [1], and MacLeod and Bucci ([12]; see also Reference [18], also provide explicit evidence of dissociations between PL and IL cortices in the contextual control of instrumental actions, acquisition of occasion-setting modulation of Pavlovian responding, and conditioned inhibition. In addition, similar effects have been found in studies using an optimal set-shifting procedure [26] designed to assess the capacity to form attentional set (as opposed to switching attentional set, cf. Reference [27]), which again found dissociations between PL and IL regions. Other recent work has also highlighted dissociations between PL and IL function (albeit using pharmacogenetic activation of local parvalbumin interneurons as a means to silence local neuronal activity [28]), although these results stand at odds with many previous findings in suggesting deficits in intra-dimensional set shifting following silencing of the PL, but *not* IL, region of the mPFC. Earlier studies found no deficit in intradimensional set shifting following extensive mPFC lesions [27], and clear evidence of set-formation deficits following IL lesions [26].

In seeking to explain the current findings, there are two possible alternatives. The first of these is that the IL and PL cortices do not have separable roles, despite previous suggestions, and instead subserve highly similar functions. However, extant literature such as that described above strongly argues against this possibility. Thus, the present findings do not discount the weight of previous evidence that, in the context of both Pavlovian and instrumental learning, PL and IL cortices subserve differential functions (e.g., References [11,29]). As such, it remains to attempt to reconcile the current results with the notion that IL and PL cortices do operate in different ways. With this in mind, a second alternative explanation is that in the current studies, lesion groups show parallel deficits, but for different reasons.

If we are to assume that the failure of PL-lesioned animals to acquire hierarchical occasion setting control over behavioural responses [11], but that the function of the IL region is complementary (and potentially opposing), then what are the options? The IL region has been implicated in the encoding of inhibition irrespective of motivational value [14], extinction of Pavlovian fear conditioning [30] as well as extinction in appetitive conditioning procedures [8,9], the development of opposing excitatory and inhibitory associations linked to the same CS [13], the ability of training to influence choice performance [1], habitual performance [20,31], and the promotion of well-learned responses over those governed by top-down control [11]. Some researchers have also highlighted the notion that the IL cortex supports behavioural flexibility in the context of extinction, but appears to oppose flexibility in the context of its promotion of extensively trained, habitual responses [32], and have proposed a role for IL in contingency tracking. In all cases, there is a link whereby the IL appears to have a central role in the development of inhibitory relationships. In the broadest context, these might be inhibitory associations between CS and US [33], encoding of CS-No US associations [34] or CS-No Event associations [35], encoding of inhibitory S-R associations [4], or bidirectional encoding of reinforced and unreinforced lever presses on an operant schedule [32]. In all cases, the IL appears to be responsible for the overlay of inhibitory or ‘no event’ relationships to existing excitatory associations or positive contingencies (see also Reference [36]). Recall also that rats lacking IL fail to show retardation in a test of inhibition that requires excitatory associations to be overlaid on existing inhibitory associations [13], suggesting this role in the encoding of opposing relationships may be bidirectional.

In the context of the current experiment, one of the requirements for the development of a hierarchical occasion setting relationship is that the target cues have a mixed history of reinforcement. It is precisely the representation of this ambiguous relationship that is modulated by the presence of the feature to afford top-down control over responding to the target. Following IL lesions, we propose that animals are no longer able successfully to encode opposing relationships or contingencies around the target cues, and therefore cannot form the type of relationship that attracts (or indeed may necessitate) the development of occasion setting. This, in turn, leaves the animals to solve the task in a manner that appears to make use of more basic associative structures, such that responding to XA− can be reduced by offsetting excitation accruing to A (following A+ trials) with inhibition to X (acquired on AX− trials); similarly inhibition accruing to B will offset excitation to Y, allowing solution of the feature positive B− YB+ discrimination. Note also that previous work has found intact summation in IL-lesioned animals, confirming that they are able to offset excitation and inhibition across separate cues [13]. Further experiments where both excitatory and inhibitory relationships are arranged with single cues would help to further elucidate this potential role of the IL.

## 5. Conclusions

Overall, the results of the current experiment then lead one to refine further the potential complementary relationship between PL and IL regions of the mPFC. Once again we have demonstrated that the PL region appears critical for the development of top-down control processes, and now further hypothesize that the IL region may be needed to represent those ambiguous relationships in the world that attract this top-down control. Of course, in many situations (and as we have shown here) animals may make use of simpler associative structures to provide behavioural solutions to non-linear discriminations, and indeed there are also configural associative structures that can provide a solution in many cases [37]. However, in order to achieve the hierarchical control that is the hallmark of truly flexible decision making, we would argue that the PL and IL must work in concert to produce the necessary template from which genuine rule-based behaviour can emerge. Understanding how rule-based behaviour is supported by these structures has the potential to open up new avenues in the treatment or prevention of human mental disorders where normal decision-making processes are disrupted, such as addiction and obsessive-compulsive disorder.

## Figures and Tables

**Table 1:** Summary of experimental design.

	Target Alone	Feature − Target	Probe
Feature Positive	B → No US	Y − B → US	Y − A → No US X − B → No US
Feature Negative	A → US	X − A → No US	

*Note*. A and B represent target stimuli (magazine light and panel light), while X and Y represent feature stimuli (tone and noise). US represents reinforcement via food pellet, and No US represents no reward. Animals are trained and tested on all combinations.
